# The Temperature-Sensitive Role of *Cryptococcus neoformans* *ROM2* in Cell Morphogenesis

**DOI:** 10.1371/journal.pone.0000368

**Published:** 2007-04-11

**Authors:** Beth Burgwyn Fuchs, Robin J. Tang, Eleftherios Mylonakis

**Affiliations:** Division of Infectious Diseases, Massachusetts General Hospital, Boston, Massachusetts, United States of America; David Geffen School of Medicine at University of California Los Angeles, United States of America

## Abstract

*ROM2* is associated with *Cryptococcus neoformans* virulence. We examined additional roles of *ROM2* in *C. neoformans* and found that *ROM2* plays a role in several cell functions specifically at high temperature conditions. Morphologically *rom2* mutant cells demonstrated a “tear”-like shape and clustered together. A sub-population of cells had a hyperelongated phenotype at restrictive growth conditions. Altered morphology was associated with defects in actin that was concentrated at the cell periphery and with abnormalities in microtubule organization. Interestingly, the *ROM2* associated defects in cell morphology, location of nuclei, and actin and microtubule organization were not observed in cells grown at temperatures below 37°C. These results indicate that in *C. neoformans, ROM2* is important at restrictive temperature conditions and is involved in several cell maintenance functions.

## Introduction

Cryptococcal infection caused by the fungal pathogen *Cryptococcus neoformans* is a global cause of significant morbidity and mortality [1]. Predisposing factors include HIV-infection, lymphoproliferative disorders, steroid therapy, organ transplantation and malnutrition [2], [3]. There has been a dramatic increase in the incidence of cryptococcosis in Africa, Thailand, and India, and in some areas cryptococcal meningitis is the leading cause of culture-positive meningitis and the leading cause of death among HIV-infected individuals with a mortality rate that can exceed 40% [2], [4], [5].

The virulence factor *ROM2* in *C. neoformans* was identified using an *in silico* approach based on its homology to *Saccharomyces cerevisiae ROM2* [6] and through a progeny-based screen for *C. neoformans* virulence factors using *Caenorhabditis elegans* as a model host [7]. A role for *ROM2* in virulence was confirmed using a mouse model [7]. In *S. cerevisiae, ROM2* plays a role in the protein kinase C (*PKC*) pathway via cell surface sensors [8]–[10] that transmit signals to Rom2p. Upon Rom2p activation by cellular stress [11], [12] it activates Rho1, as a guanyl nucleotide exchange factor (GEF), exchanging a GDP for a GTP [13]. In *S. cerevisiae*, Rho1 binds and activates the Pkc1 protein kinase, which in turn activates a MAPK module; Pkc1 phosphorylates Bck1, a MAPK kinase kinase, which transmits the signal to both MAPK kinases, Mkk1 and Mkk2. The PKC pathway activated by Rho1 is involved in actin cytoskeleton organization and the transcription of cell wall biosynthesis genes [14]–[17] and Rho1 controls cell wall synthesis directly by activating the (1,3)β-glucan synthase *FKS1* [18], [19]. Finally, Rho1 interacts with the protein Bni1p which has also been implicated in actin organization and is involved in polarized exocytosis [20]–[24].

Because an initial analysis indicated that *ROM2* is involved in cell growth specifically at high temperature conditions and that the *rom2* mutant is hypersensitive at high temperature conditions [7], we further examined the temperature sensitive phenotype related to virulence and cell morphology. We confirmed that the role of *ROM2* is temperature sensitive. *ROM2* was involved in actin and microtubule organization. Changes to cytoskeletal components were coupled with changes in cell morphology and cell separation defects. These defects were not observed in cells grown at temperatures below 37°C.

## Results and Discussion

### *ROM2* is involved in *C. neoformans* morphogenesis

**Figure 1:**
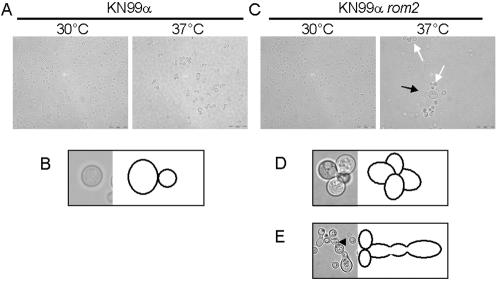
Micrographs and diagrams of *C. neoformans* morphology after 24 hours of growth are presented for KN99α and KN99α *rom2*. A. KN99α cells grown at 30°C and 37°C. Scale bars represent 20 µM. B. The KN99α cells appear to have a round morphology. The left image is a micrograph and the right image is a diagram. C. KN99α *rom2* cells grown at 30°C and 37°C cluster together. At 37°C growth conditions, the hyperelongated morphology is also observed. White arrows indicate cells that are “tear” shaped and form a cluster. The black arrow indicated a hyperelongated complex. 100× magnification. D. KN99α *rom2* cells appear as a cluster of 3–4 cells per complexes. The left image is a micrograph and the right image is a diagram. E. KN99α *rom2* cells also demonstrate a hyperelongated phenotype at 37°C that forms a chain of cell complexes. The arrowhead indicates the region of the hyperelongated cell defective for cytokinesis, indicated by the lack of cell wall formation between to cell complexes. The left image is a micrograph and the right image is a diagram of the cell morphology.

An evaluation of the cell morphology at 30°C and 37°C growth temperatures established KN99α *rom2* has a morphological defect at 37°C growth conditions (**Figure 1A and B**). More specifically, we found that the KN99α *rom2* cells were slightly elongated or “tear” shaped rather than the normal round shape of wild type *C. neoformans* (**Figure 1C and D**). A state of elongation has been observed previously for *rom2* mutants in *S. cerevisiae* [25]. The “tear” shaped structure was enlarged at one end of the cell but smaller at the other polar end of the cell and connected to other cells with the same morphology. The mutant cells did not form a chain but connected at a common point amongst the cells (**Figure 1D**). In addition to the clustered “tear” shaped cells there was also an additional phenotype found in a sub-population of cells characterized by a hyperelongation (**Figure 1E**).

### Hyperelongated morphology and defects in nuclear localization

The hyperelongated structures were many times longer and several times wider than the diameter of normal, unbudded cells. The cells of the complex formed a chain and cells within the chain varied in size. A visible cell wall was often not detected between newly budded cells indicating a lack of cytokinesis (**Figure 1E**).

After 24 h of growth at 37°C, 4.1%±0.6% of KN99α *rom2* cells formed a hyperelongated structure. The hyperelongated structure was not found for KN99α *rom2* cells at 35°C (or lower). The presence of a minor population of elongated cells is consistent with the elongated buds found to comprise 7% of the cell morphologies in the *S. cerevisiae rom2* mutant [25]. We describe the KN99α *rom2* phenotype as hyperelongated rather than the elongated term used to describe the *S. cerevisiae rom2* mutant because of the cell elongation coupled with the lack of cell separation in this sub-population of cells.

**Figure 2:**
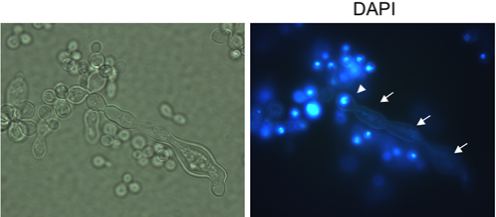
Nuclear defects in hyperelongated cells at high temperature conditions. The light field image on the left shows a hyperelongated cell. The image on the right is a DAPI staining of the same hyperelongated cell complex. The arrowhead indicates the location of the nucleus. Arrows indicate the cell complexes that lack a nucleus.

Due to cell separation defects, we looked for changes in nuclear location in *C. neoformans* cells using 4′,6-diamidino-2-phenylindole dihydrochloride (DAPI) staining. Interestingly, hyperelongated cells that are present at high temperatures show defects in the location of nuclei. Hyperelongated cells contain multiple complexes in a chain and many of the complexes were absent of a nucleus (**Figure 2**). This indicates that the nucleus is not properly dividing or the complexes are not true buds. Overall, the lack of nuclei in the hyperelongated complexes indicates a role for *ROM2* in nuclear division or proper localization of nuclei in the budded cells. The lack of nuclear material in complexes of the hyperelongated cells may also be associated with the cytokinesis defects between the cell complexes of the hyperelongated *rom2* mutant cells.

### Role of *ROM2* in actin polarization

The cytoskeleton is the infrastructure of the cell that determines and maintains cell shape. Two major components of all eukaryotic cytoskeletons are microfilaments, composed of actin subunits, and microtubules, made up of tubulin heterodimers. The actin cytoskeleton plays an important role in maintaining cell morphology during growth and cell division of yeast cells [26]. The location of actin has suggested roles in the movement of secretory vesicles and anchoring of the actin cytoskeleton to the cell membrane [26], [27]. The actin cytoskeleton also polarizes the secretory apparatus to the daughter cell to provide the materials needed for the assembly of the cell wall [28].

The normal distribution of F-actin cytoskeleton in *C. neoformans* has been examined previously [29] and is comprised of cortical patches, cables and cytokinetic rings. In *S. cerevisiae*, cortical patches are concentrated at areas of bud formation and fibers occur along the axes of the mother-daughter pair [26]. In *C. neoformans*, cortical patches are evenly distributed throughout the cell and actin cables are directed to the growing daughter cell [29]. In general, actin is distributed in areas of cell growth for *C. neoformans* and *S. cerevisiae* [26], [29].

*ROM2* was previously shown to be involved in cytokinesis and morphogenesis under stress conditions in *S. cerevisiae* [25]. In *S. cerevisiae*, Rom2p is concentrated at sites of polarized growth and co-localizes with actin at the site of bud emergence [25]. Depolarized actin has been shown to cause an abnormal cell shape and with the Rho family of GTPases [24], [30]–[34]. A similar defect has been shown to cause abnormal morphology in *C. neoformans ras* mutants [35]. We hypothesized that a potential cause in the morphological changes of the *rom2* mutant, are changes in actin polymerization and organization. Therefore, we assessed actin localization of *C. neoformans* KN99α *rom2* using rhodamine-phalloidin that stains for F-actin.

**Figure 3:**
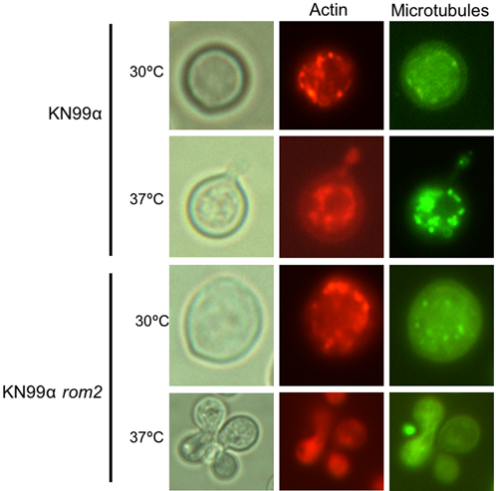
Individual cell localization of actin and microtubules. Actin staining with rhodamine-phalloidin of both KN99α and KN99α *rom2* cells at 30°C and 37°C. Microtubules were identified using a TAT1 antibody and staining with goat anti mouse secondary antibody as described in the materials and methods.

**Figure 4:**
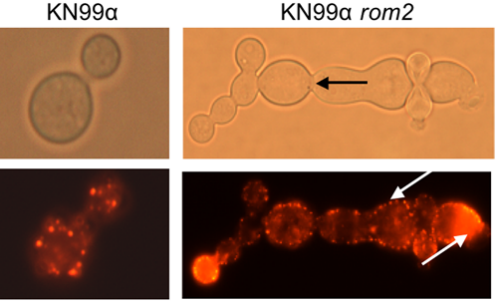
*C. neoformans* actin expression at 37°C in KN99α and KN99α *rom2*. F-actin is identified with rhodamine-phalloidin. Actin is distributed throughout the wild type cell. White arrows indicate actin localization at the cell periphery and at the bud tips of KN99α *rom2* hyperelongated cells. The black arrow indicates the region of the cell that is defective for cytokinesis indicated by a lack of cell wall formation between the cell complexes.

At 30°C, staining with rhodamine-phalloidin showed actin concentrated in cortical patches evenly distributed throughout the cell of KN99α *rom2* at comparable density to wild type for KN99α *rom2* budded or unbudded cells (**Figure 3**). Polarization was observed in budding cells for both KN99α and KN99α *rom2* at 30°C and 37°C. Interestingly, in the KN99α *rom2* hyperelongated cells, actin was located at the cell periphery rather than being distributed throughout the cell (**Figure 4**). Actin was highly concentrated at the bud tips of the growing KN99α *rom2* hyperelongated cells. The concentration of actin at the cell wall indicates either continued growth of the cell or movement of cell wall components for cell wall repair [36].

### Role of *ROM2* in microtubule organization

In *S. cerevisiae*, there is evidence that links *ROM*2 to the nuclear and cytoskeletal organization of microtubules. In yeast cells microtubules facilitate the migration of nuclear material. The *S. cerevisiae ROM2* homologue is an upstream activator of Rho1 and the Rho1 downstream target Bni1p has been suggested to play a role in microtubule-related nuclear migration [37]. More specifically the *ROM2* role in nuclear organization was demonstrated when *S. cerevisiae ROM2* was shown to rescue the microtubule mutants *cik1*and *kar3* which are involved in spindle pole assembly and localized to the spindle pole body (SPB) and cytoplasmic microtubules [25], [38]. The ability of *ROM2* to suppress these mutants in *S. cerevisiae* indicates a possible role in the motor function of microtubules [38]. The role of *ROM2* in microtubule organization is further indicated by *S. cerevisiae rom2* mutant sensitivity to the microtubule depolymerizing drug benomyl [25]. However, the *S. cerevisiae rom2* mutant did not demonstrate visible signs of microtubule defects [25].

**Figure 5:**
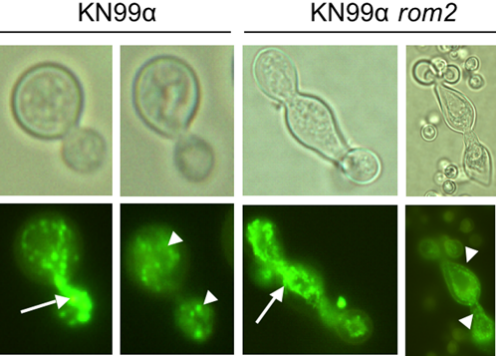
Microtubule localization at 37°C conditions. Microtubules are distributed throughout wild type cells. Microtubules are found in localized areas of KN99α *rom2* hyperelongated cells. The distribution of cellular material between mother and daughter cell is well defined for wild type cells but is enlarged over a greater area in hyperelongated cells. Arrowheads indicate the location of microtubule patches. Microtubules are distributed throughout mother and daughter cells in KN99α. However, microtubules are localized to the neck region and periphery of the hyperelongated KN99α *rom2* cells. Arrows indicate microtubule extensions between mother and daughter cells.

Unlike the *S. cerevisiae rom2* mutant, microtubule defects in the *C. neoformans rom2* mutant were visible. We evaluated the role *ROM2* in *C. neoformans* microtubule organization using a TAT1 antibody [39]. Our studies indicate that in the hyperelongated phenotype microtubules, like actin, are also located at the cell periphery (**Figure 3**). The microtubule location suggests defects in the cell wall and repair which were also suggested for actin [36]. More specifically, we found that microtubules were extended into the neck between the mother and daughter cell during budding for both KN99α and KN99α *rom2*. Microtubules also formed patches located throughout the cell (**Figure 5**). However, in the case of hyperelongated cells that formed a cluster or chain of cell complexes, microtubules extended between the mother and daughter cells and appeared larger than the extensions between KN99α mother and daughter cells (**Figure 5**). While filamentous tubulin structures were evident in most wild type cells they were not always evident in KN99α *rom2* cells (**Figure S1**).

**Figure 6:**
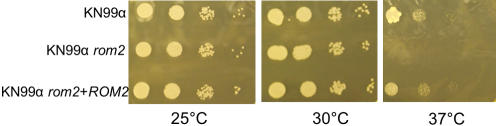
Sensitivity to nocodazole was assessed using KN99α, KN99α *rom2*, and KN99α *rom2*+*ROM2*. Cultures were grown overnight and serial dilutions were plated on YPD with 2 µM nocodazole. Plates were grown for 2 days at 25°C, 30°C or 37°C to detect growth inhibition.

Microtubule defects were confirmed by hypersensitivity to nocodazole, which depolymerizes microtubules by binding to β-tubulin and interfering with microtubule assembly. KN99α *rom2* cells failed to grow in the presence of 0.2 µM nocodazole (**Figure 6**). Interestingly, the hypersensitivity of KN99α *rom2* to nocodazole was a temperature sensitive effect at the restrictive temperature of 37°C. Wild type *C. neoformans* were sensitive to nocodazole at concentrations greater than 0.2 µM (data not shown).

In conclusion, there are several temperature sensitive phenotypes associated with *ROM2*. *ROM2* plays a temperature sensitive role in actin and microtubule organization. The actin and microtubule defects associated with the *rom2* mutant are most visible in the minor population of hyperelongated cells that are formed at high temperature conditions. These actin and microtubule organization defects are coupled to defects in cell morphology, indicated by the hyperelongated phenotype, and defects found for the distribution of nuclear material. Thus the temperature sensitive role of *ROM2* is important for several cell maintenance functions of *C. neoformans*.

## Materials and Methods

### Strains and media

*C. neoformans* strains used in this study include KN99α, KN99α *rom2* and the reconstituted strain KN99α *rom2*+*ROM2* [7]. *C. neoformans* cultures were grown using YPD (1% yeast extract, 2% peptone and 2% dextrose) media. Cultures were grown at 30°C unless otherwise specified. Cells were grown in liquid culture for assays observing cell morphology.

### Fixation and staining of actin, microtubules and nuclei

Cells were fixed and stained for F-actin according to the protocol by Kopecká and colleagues (2001) [29] with an increased permeabilization time of 15 minutes. In brief, cells were grown in liquid culture overnight at 30°C and 37°C. Then cells were washed twice with wash buffer (0.1 M KH_2_PO_4_, 1.25 mM EGTA, 1.25 mM MgCl_2_ at pH 6.9) for three minutes. Cells were suspended in 100 uL of fixative (5% paraformaldehyde in wash buffer), incubated at room temperature for 90 minutes then washed three times with wash buffer for five minutes. Cells were treated with *Trichoderma harianum* lysing enzyme (Sigma) (1 mg ml^−1^) for 20 minutes at room temperature. Cells were collected with centrifugation then permeabilized by adding 0.3% Triton X-100 for 15 minutes at room temperature. Triton X-100 was removed and cells were suspended in 2% bovine serum albumin (BSA) in phosphate buffer saline (PBS) at 37°C for 30 minutes. The BSA was removed and a 1∶100 dilution of TAT1 monoclonal antibody [39] in PBS was added and incubated at 37°C for 60 minutes [39]. Cells were collected with centrifugation and washed three times with PBS for five minutes. Cells were then suspended in 25 uL (5 U) of rhodamine-phalloidin to identify F-actin and 100 uL of 1∶200 diluted goat anti-mouse Flour 488 (Invitrogen) as a secondary antibody to identify TAT1 for 90 minutes at 37°C. Cells were also stained with 0.1 µg ml^−1^ DAPI for 15 minutes. Cells were collected then washed twice with 1% BSA in PBS for five minutes followed by a five minute rinse in 0.1% BSA in PBS. Cells were suspended in Vectashield (Vector Laboratories, Inc.) and visualized with an Olympus microscope. All observations were confirmed with three independent cell cultures.

### Nocodazole

Ten fold serial dilutions from 10^4^ to 10^1^ of KN99α, KN99α *rom2* and KN99α *rom2*+*ROM2* cells were plated in a volume of 5 µL on YPD containing 0, 0.1 µM, or 0.2 µM nocodazole (Sigma) from a stock concentration of 100 µg ml^−1^ nocodazole dissolved in DMSO. Plates were incubated for 2 days at 25°C, 30°C, and 37°C to allow colonies to grow.

## Supporting Information

Figure S1 Microtubules are observed in budding cells directing toward the emerging bud. Although microtubules were observed in KN99α *rom2* cells the microtubules were less frequent and more diffuse throughout the cell.
(0.52 MB TIF)
