# Supplementary figures and images for: The Temperature-Sensitive Role of Cryptococcus neoformans ROM2 in Cell Morphogenesis
Source: PLoS One. 2007 Apr 11;2(4):e368. doi: 10.1371/journal.pone.0000368 (PMC1838519; doi:10.1371/journal.pone.0000368)

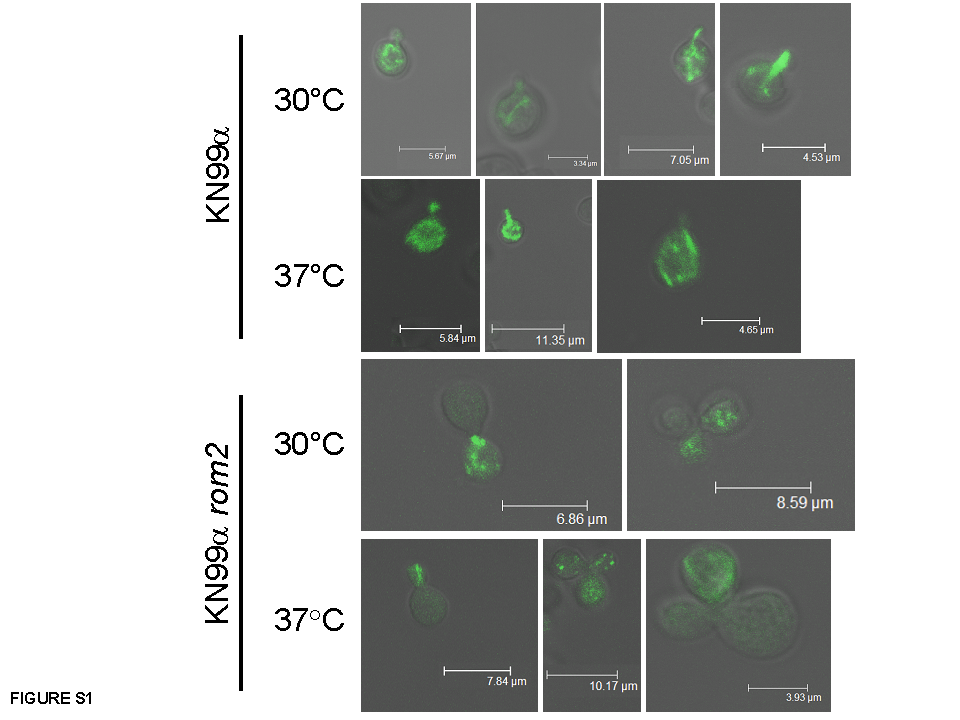

Supplement: Figure S1 — Microtubules are observed in budding cells directing toward the emerging bud. Although microtubules were observed in KN99α rom2 cells the microtubules were less frequent and more diffuse throughout the cell. (0.52 MB TIF) [file pone.0000368.s001.tif]
